# PD175 Using Machine Learning To Optimize Systematic Literature Reviews

**DOI:** 10.1017/S026646232400401X

**Published:** 2025-01-07

**Authors:** Akshay Chacko

## Abstract

**Introduction:**

Screening and selecting publications are very time consuming when conducting systematic literature reviews. Currently, in the field of robotic-assisted surgery (RAS) there is an average of 12 to 15 studies published daily, making manual data management unsustainable. We aimed to investigate how machine learning (ML) can be used to optimize the manual processes of literature reviews.

**Methods:**

New RAS publications in PubMed, Scopus, and Embase are routinely screened for relevancy and then tagged with metadata to aid future analysis. A curated library of approximately 40,000 tagged RAS publications served as our training dataset. To support manual screening and tagging efforts, multiple ML models were benchmarked, including logistic regression, decision trees, and gradient boosting. All model implementations came from the Python scikit-learn package. The evaluation metric for this study was the F1 score, and the fields of interest tagged were procedure type and surgical approach. Models were trained on publication abstracts and compared with a baseline keyword search to measure changes in performance.

**Results:**

The findings demonstrated that ML models can classify key metadata with high levels of accuracy. The decision tree model correctly labeled the five most common procedures in the dataset, with an average F1 score of approximately 0.90. This same model predicted surgical approach with an average F1 score of 0.84. It is important to note that different models performed best in different scenarios. To compensate for this variability, all models were fed into a stacking classifier—an ensemble model that takes the output of other models as input training data.

**Conclusions:**

It is evident that ML models can reduce the cognitive burden of clinical librarians and shift their role from hand-screening papers to validating ML predictions. Future work may involve comparing the performance of traditional ML models with large language models (LLMs) to further improve F1 scores and reduce class imbalances.

